# The characteristics of users of an online screening tool for children and adolescents with intellectual disability and of those being screened

**DOI:** 10.1177/17446295241272698

**Published:** 2024-08-08

**Authors:** Karen McKenzie, Kara R. Murray, Judith Thompson, Karen Horridge, Kirsty Greenwell, Aja L. Murray

**Affiliations:** Department of Psychology, 5995 Northumbria University, UK; Community Mental Health, 3129 NHS Lothian, UK; North East and Cumbria Learning Disability Network, UK; Department of Education, 7735 University of Sunderland, UK; North East and Cumbria Learning Disability Network, UK; Department of Psychology, University of Edinburgh, UK

**Keywords:** intellectual disability, child and adolescent intellectual disability screening questionnaire, characteristics

## Abstract

Screening tools can help with the identification of intellectual disability, but little is known about who uses them. This study analysed anonymous information from 2691 users of an evidence-based, online, intellectual disability screening questionnaire for children and adolescents (CAIDS-Q) to explore the characteristics of the users and of those being screened. The users were split almost equally between parents/family members (48.6%) and professionals (49.9%), with the majority (63.8%) of the latter group being health staff. Significant differences in the characteristics of the children being screened were found, according to whether the user was a parent/family member or a professional, with the overall pattern suggesting that professionals screened children with greater complexity of needs, but about whom less was known. The screened children had a range of areas of difficulties that are common to those with intellectual disability. Implications for practice are discussed.

## Introduction

Many parents of children with developmental disabilities face challenges when seeking formal identification of their child’s condition. This can include lengthy waits for assessment, difficulties with navigating what can be complex service processes and a growing sense of urgency based on the awareness that receiving support and intervention for their children at as early a stage as possible is crucial (Rivard et al., 2021). Much of the research in this area relates to parents of autistic children, but studies suggest that the process of seeking a diagnosis can also be negative and stressful for parents of children with an intellectual disability (Watson et al., 2011).

There are a number of reasons why the identification of intellectual disability can be difficult. Morin and colleagues (2022) provide an overview of some of the systemic, family and child characteristics that have been found to be related to delays in, and parental dissatisfaction with, the identification processes for children with developmental disabilities. Systemic factors, such as long waiting times for referral and assessment, combined with the complex and time-consuming nature of the assessment processes can present barriers to early identification and intervention for the children and reduce parental satisfaction with services. Research with parents of autistic children suggest that family characteristics, such as ethnic and national origin can also influence both the extent of delays in identification and parental satisfaction. In this context, research has found that those children whose parents were born in a country other than the one in which they now live were more likely to be older at the point of referral for assessment than children of parents from the same country (Valicenti-McDermott et al., 2012). In addition, South-East Asian parents/carers of children with an intellectual disability who do not speak English or have English as a second language have also been found to engage less with services, indicating that language is a significant barrier to access (Robertson et al., 2019).

Morin et al. (2022) note that the relationships between child characteristics, delays in identification and parental satisfaction is complex, with researchers finding that both milder and more severe symptoms of the child may be associated with greater parental satisfaction with the identification process. The latter result is suggested to be due to a shorter delay in identification. Many of the studies outlined by Morin et al. (2022), however, relate to autistic children and there is a need for specific research with those with intellectual disability.

It is recognised that screening tools can have a role in helping children with developmental disabilities to be identified (American Academy of Pediatrics Committee on children with disabilities, 2001), for example by helping professionals to prioritise those who would most benefit from further assessment (British Psychological Society, 2015). A number of screening tools exist that screen for general developmental disabilities or specific conditions such as autism (see Marlow et al., 2019 for a review). There are, however, very few evidence-based screening tools that are specifically designed to screen for intellectual disability and which cover a younger age group. For example, while the Hayes Ability Screening Index (Hayes 2000), was developed to screen for intellectual disability, it is only suitable for people aged 13 years and over.

In addition, many screening tools are designed to be used by clinicians or specific professional groups (Marlow et al., 2019), meaning that parents and family members would be unable to access them. By contrast, the Child and Adolescent Intellectual Disability Screening Questionnaire (CAIDS-Q^:^ McKenzie et al., 2012) was specifically designed to screen for intellectual disability in those aged 6 years to just under 18 years, and to be accessible to a range of people – in particular the user does not need to be a professional or to have a particular qualification or training. Previous research has also suggested that the use of the CAIDS-Q can result in reported benefits such as validating concerns that people may have about a child; speeding up the identification process; increasing understanding and acceptance of the child; increasing awareness of intellectual disability; helping the person and family to get extra support; and importantly, identifying those whose intellectual disability was previously unknown (McKenzie et al., 2019, 2021).

In order to further increase its accessibility, in 2020 the CAIDS-Q was included as part of a suite of tools available via the Learning Disability Matters for Families website (see https://learningdisabilitymatters.co.uk/), enabling it to be easily accessed by both parents and professionals. This provided the chance to explore the characteristics of those using the CAIDS-Q and of the children and adolescents about whom the CAIDS-Q had been completed. Based on the research outlined by Morin et al. (2022), in relation to children with autism, it might be expected that some of these characteristics might differ depending on whether the screening tool was completed by parents or professionals.

## Aim

The research aimed to analyse the information provided by users of the online CAIDS-Q to address the following questions: who uses the online screening tool; what are characteristics of the children who have been screened and; do their characteristics differ according to whether the user was a parent or professional?

## Method

### Design and ethics

The study was granted ethical approval by the first author’s university ethics committee. A quantitative design was used. Anonymous data that was generated automatically when a user completes the CAIDS-Q was analysed. Users provide digital consent to the use of the data for research purposes in line with the privacy policy of the website.

### Participants

Participants were people who had completed the online CAIDS-Q in relation to 2691 children or adolescents aged from 4 to 18 years.

### Materials

Data were obtained from responses to the CAIDS-Q items, basic demographic information and information that was gathered because it may influence the outcome of the screening tool e.g., whether the person speaks English as a second language (see https://learningdisabilitymatters.co.uk/tools/ and Table 1 for details of questions). The online system uses the responses to calculate the person’s score. Feedback, based on an evidence-based cut-off score that takes account of the child’s age, is provided to the user, along with guidance on sources of support and information. The online version of the CAIDS-Q has been shown to have good test-retest reliability, and high sensitivity values (McKenzie et al., 2020, 2023).

**Table 1.** Characteristics of the child/young person for the whole sample, parent/family users and professional users, with comparisons of the latter two groups.

Characteristic	Whole sample (n = 2691)		Parent/family users only (n = 1307)		Professional users only (n = 1342)		Comparison between Parent/family and professional users	Significance value
Demographic information	Range	Mean (SD)	Range	Mean (SD)	Range	Mean (SD)		
Age	4-18 years	10 years 9 months (3 years, 6 months)	4-18 years	10 years, 8 months (3 years, 7 months)	4.5 -18 years	10 years, 9 months (1 month)	t = .590	.555
	**Number (%)**		**Number (%)**		**Number (%)**			
Gender	**Male**	**Female**	**Male**	**Female**	**Male**	**Female**	χ2 = 1.09	.296
	1574 (58.5)	1117 (41.5)	754 (57.7)	553 (42.3)	801 (59.7)	541 (40.3)		
	**Number (%)**		**Number (%)**		**Number (%)**			
English as a second Language	430 (16)		200 (15.3)		221 (16.5)		χ^2^ = .673	.412
**Academic Issues**								
Able to read	1581 (58.8)		849 (65)		707 (52.7)		χ^2^ = 41.17	**<.001**
Able to write	1060 (39.4)		554 (42.4)		488 (36.4)		χ^2^ = 10.07	**.002**
Able to tell the time	811 (30.1)		441 (33.7)		350 (26.1)		χ^2^ = 18.55	**<.001**
Receives support at school	2013 (74.8)		950 (72.7)		1036 (77.2)		χ^2^ = 7.19	.007
**Self-care skills**								
Able to use toilet independently	1992 (74)		956 (73.1)		1004 (74.8)		χ^2^ = .959	.328
Able to tie laces	1044 (38.8)		515 (39.4)		505 (37.6)		χ^2^ = .879	.349
Able to make a simple meal	1553 (57.7)		812 (62.1)		719 (53.6)		χ^2^ = 19.84	**<.001**
**Relationship skills**								
Able to recognise emotions	1509 (56.1)		795 (60.8)		691 (51.5)		χ^2^ = 23.43	**<.001**
Has one or more friends	1789 (66.5)		893 (68.3)		868 (64.7)		χ^2^ = 3.95	.047
**Health issues**								
Previous/current contact with intellectual disability services	542 (20.1)		321 (24.6)		210 (15.6)		χ^2^ = 32.91	**<.001**
Reported intellectual disability	576 (21.4)		324 (24.8)		250 (18.6)		χ^2^ = 14.81	**<.001**
Reported autism	823 (30.6)		470 (36)		343 (25.6)		χ^2^ = 33.68	**<.001**
Reported learning difficulty e.g., dyslexia	794 (29.5)		403 (30.8)		379 (28.2)		χ^2^ = 2.14	.144
Reported other condition e.g., physical disability	468 (17.4)		268 (20.5)		193 (14.4)		χ^2^ = 17.3	**<.001**
No reported condition	814 (30.2)		336 (25.7)		462 (34.4)		χ^2^ = 29.9	**<.001**
Identified by CAIDS-Q as likely to have an intellectual disability	1720 (63.9)		791 (60.5)		907 (67.6)		χ^2^ = 14.4	**<.001**
	**Range**	**Mean (SD)**	**Range**	**Mean (SD)**	**Range**	**Mean (SD)**		
Number of reported conditions	0-5	.99 (.88)	0-5	1.14 (.94)	0-4	.873 (.799)	t = 7.745	**<.001**
Total CAIDS-Q score	0-7	3.4 (1.8)	0-7	3.5 (1.8)	0-7	3.2 (1.8)	T = 3.843	**<.001**

### Procedure

The information from the online tool was exported into an excel spreadsheet. Data that was entered during the testing phase of the online CAIDS-Q was removed. There were some children whose ages were recorded as falling outside the age range for which the CAIDS-Q was validated. The data for these children was retained, as the purpose of the study was to explore the characteristics of users and the children being screened. SPSS was used to analyse the final dataset. Descriptive statistics were used to describe the characteristics of the whole sample and separately according to whether the user was a parent or professional. A series of chi-square tests were used for dichotomous data (e.g. gender) and t-tests for continuous data (e.g. age) to determine if any of the child characteristics differed according to whether the user was a parent or professional. A Bonferroni correction was used which changed alpha from 0.05 to 0.0026 to adjust for multiple comparisons. While this approach is considered by some researchers to be too conservative and risks missing genuinely significant results, it has the advantage of minimising falsely significant findings (VanderWeele & Mathur, 2019).

## Results

### Who uses the screening tool?

In total, 1307 (48.6%) of those using the online version of the CAIDS-Q were parents or family members of the person being screened, 1342 (49.9%) were professionals (e.g., health or education staff) and 42 (1.6%) classified themselves as ‘other’ e.g., researcher. The professional group could be broken down further into education staff (n = 374), health staff (n = 856), other professional, e.g., social worker (n = 73) and not specified (n = 39).

### What are characteristics of the children who have been screened?

Table 1 shows the characteristics of the children and adolescents about whom the CAIDS-Q was completed. This is provided for the total sample, and for parent/family and professional users.

### Do the characteristics of the children being screened differ according to whether the user was a parent or professional?

Table 1 shows the results of comparisons between the parent and professional users of the online screening tool. This illustrates significant differences between the two groups. Children who were screened by users who were professionals had an overall lower total CAIDS-Q score, indicating increased likelihood of intellectual disability. They were less likely to be able to read, write, and tell the time, to make a simple meal, such as a sandwich, and recognise emotions as compared with those screened by parent/family member users. In respect of health, children who were screened by professional users were less likely to have had previous contact with intellectual disability services and less likely to have a reported intellectual disability, autism, or other condition, as compared with those screened by parent/family member users.

Children screened by professional users were also more likely to have no reported existing condition, to be identified by the CAIDS-Q as likely to have an intellectual disability and to have a lower mean total number of reported conditions as compared with those screened by parents/family members.

## Discussion

The study aimed to address three main questions. The first related to who was using the online version of the screening tool. The results indicated that the users were nearly equally split between parents/family members and professionals, with health staff comprising the largest percentage of the latter group. The inclusion of the CAIDS-Q in the ‘Learning Disability Matters for Families’ website was designed to give parents and carers of children with an intellectual disability (or with concerns that their child might have an intellectual disability) increased access to an evidence-based screening tool. This appears to have been successful, with nearly half of the CAIDS-Q users being parents or family members. The online version of the screening tool may also offer the advantage of providing feedback from the CAIDS-Q in the context of additional online guidance about potential next steps following screening. It links to both general and local sources of support, depending on the area that the user comes from. As the CAIDS-Q is hosted on the Learning Disability Matters for Families website, users can also easily access further information, guidance, and links to support from the website itself.

A range of professionals were also making use of the screening tool, with the largest group being health professionals. The hard copy versions of the CAIDS-Q and the adult equivalent, the LDSQ have previously been found to be used by a wide range of professionals in many different contexts (McKenzie et al., 2021) and the current research suggests that a similar range of professionals are using the online version.

The second research question explored the characteristics of those being screened. Overall, there were more males than females being screened. This may reflect the fact that intellectual disability is more commonly identified in males than females (American Psychiatric Association, 2023). The data showed that 16% of the children overall and 15.3% of those screened by parents spoke English as a second language, indicating that they or their family may originate from a country other than the UK. While equivalent UK population figures are not available for the specific age range of children who were screened, the Census (2021) indicated that 8.9% of the population of England and Wales spoke English (or Welsh) as a second language. This suggests that the online CAIDS-Q may offer an accessible way for parents whose main language is not English to obtain an indication of whether their child is likely to meet the criteria for intellectual disability or not. This is important as language barriers have been found in previous studies to reduce engagement with services (Robertson et al., 2019). Translating the CAIDS-Q into other languages in future could increase its accessibility further.

People with an intellectual disability, by definition, have significant difficulties with their cognitive and adaptive skills (World Health Organization, 2022) and it might be expected that many of the children who were being screened would have difficulties in at least some of the areas being measured. Overall, the characteristics of the children being screened suggest that this is the case. For example, less than half were reported as being able to write or tell the time and nearly three quarters received support at school. None of the skills were able to be completed by all the children. While this would be expected in relation to some skills because of the ages of the children, for example it would not be expected that all six year-old children could tell the time, other skills would be achievable for most typically developing children at that age, for example identifying basic emotions (Grosse et al., 2021).

Nearly a third of the children being screened were reported as being autistic. Intellectual disability and autism commonly co-occur, with up to 70 % of autistic people being estimated to also meet the criteria for intellectual disability (Buescher et al., 2014, Matson & Shoemake, 2009). There is also overlap in some of the indicators of both conditions (Thurm et al., 2019), such as difficulties with emotion recognition and friendships. Research has suggested, however, that the CAIDS-Q can accurately identify intellectual disability in autistic people, with sensitivity values of 93.1% (McKenzie et al., 2023a) and 92% (McKenzie et al., 2023) for the hard copy and online versions respectively.

The third research question explored whether there were any significant differences in the characteristics of the children who were screened by parents/family members, as compared with professionals. The results indicated a number of differences. In respect of academic skills, the professionals were screening children who were more likely to have difficulties with their literacy and numeracy skills than those screened by parents/family members. Research suggests that difficulties with reading and writing (Wakeman et al., 2021) and comprehension of many of the abstract principles that underpin basic numeracy (Park et al., 2020), are relatively common in people with an intellectual disability. As the ability to read, write and tell the time is essential to many activities, poor literacy skills can act as a barrier to fully engaging in daily life.

In terms of wider adaptive and relationship skills, the children who were screened by professionals were also found to be less likely to be able to make a basic meal, such as a sandwich, and to be able to recognise emotions. Research has shown that children with an intellectual disability are more likely to have difficulty with emotion recognition than their peers who do not have an intellectual disability (Murray et al., 2019). Such difficulties have been found to be associated with poorer social outcomes, from being perceived as less capable in social situations to rejection by other children (Leppanen & Hietanen, 2001 Mostow et al., 2002).

The differences in the characteristics of the children screened by professionals as compared with parents represent areas of difficulty which are commonly experienced by those with an intellectual disability, and which can have a significant detrimental impact. As such, they may have acted as indicators to the professionals that the child may be experiencing developmental difficulties, which in turn prompted the professional to screen for intellectual disability in order to have a greater understanding of the child’s needs.

This suggestion is supported by the fact that the children who were screened by professional users of the CAIDS-Q were less likely to have had previous contact with intellectual disability services or to have existing identified conditions (with the exception of specific learning difficulties). They were also more likely to have a lower total CAIDS-Q score, feedback based on their CAIDS-Q score of being likely to have an intellectual disability, and to have a lower overall mean number of reported conditions.

This suggests that the professionals were screening children who had greater complexity of needs in terms of their day to day adaptive and cognitive functioning, but about whom less was known. The results overall could be interpreted in the context of the challenges that parents face in having their child’s developmental disability identified (Rivard et al., 2021, Watson et al., 2011) and shortages of professionals who can conduct relevant clinical assessments (British Medical Association and the Association of Clinical Psychologists, 2020). The children who come to the attention of professionals in the first place are likely to have more complex and as yet unidentified needs. It may be that professionals are using the outcome of the screening tool to help with decisions about who to offer further assessment to, or to refer on for further assessment in the context of high demand for services and shortages of clinical staff (British Psychological Society, 2015). This interpretation is lent some support from previous research, in which 67% of participating clinical staff agreed that using the CAIDS-Q helped the service to prioritise diagnostic assessment (McKenzie et al., 2019).

The results could also suggest that some parents may be using the screening tool because they have some concerns that their child’s existing identified condition does not fully explain their difficulties. For those who have a reported condition other than intellectual disability, the parents may be exploring whether their child’s profile could be better explained by intellectual disability instead of, or in addition to their existing condition. This may particularly be the case with parents of autistic children, given the overlap in some behavioural characteristics with intellectual disability (Thurm et al., 2019). While the motivation for using the CAIDS-Q is unknown, previous research with parents has found that the majority agreed that using the CAIDS-Q helped identify children who were likely to have an intellectual disability and who were potentially vulnerable, as well as increasing understanding of their difficulties and support needs (McKenzie et al., 2019). Further research that explores the ways in which users of the online version of the CAIDS-Q tool plan to use the outcome will help clarify this.

### Strengths and limitations

A major strength of the study was that it had a large sample size and was based on data that was gathered from people in a naturally occurring, rather than a research context. As such, it is likely to reflect users who felt that the child or adolescent being screened had difficulties that may be consistent with intellectual disability.

In respect of imitations, in common with other informant-based measures, the data used in the study was based on user reports, rather than being based on observation or assessment with the child. In addition, because the online version of the CAIDS-Q was designed to be quick and easy to complete the number of questions which were included in addition to the core CAIDS-Q items was limited. There may be other important differences between parent and professional users that were not identified by the existing questionnaire, for example, the motivation for completing the CAIDS-Q and how the results will be used. Future research would be needed to address such questions. Finally, as the CAIDS-Q was hosted on a UK website and signposted users to UK resources, it is likely that most lived in the UK. Obtaining information about the country of origin of the users in future research would help clarify whether the results found in the present study are also applicable to other countries.

### Conclusion

The results of the study suggest that the online version of the screening tool is being used by a wide range of parents and professionals in relation to children who have difficulties in some areas of their cognitive and/or adaptive skills. The study illustrated that the CAIDS-Q users are broadly split between parents/family members and professionals. A number of significant differences were found in the characteristics of the children who were screened by parent/family members compared with professionals. Overall, the differences suggested that the children being screened by professionals may have more complex needs and have less known about them in terms of pre-existing conditions or contact with intellectual disability services.

## Data Availability

The data that support the findings of this study are available from the corresponding author upon reasonable request. *
